# Critical Contextual Empiricism for Busy People: Scientific Argumentation as Epistemic Exchange

**DOI:** 10.1007/s11245-025-10198-0

**Published:** 2025-04-16

**Authors:** Catarina Dutilh Novaes, Çağlar Dede

**Affiliations:** 1 VU Amsterdam, Amsterdam, Netherlands; 2 Independent Scholar, Utrecht, Netherlands

**Keywords:** Scientific argumentation, Critical contextual empiricism, Peer review, Epistemic exchange

## Abstract

In her account of science known as *critical contextual empiricism* (CCE), Helen Longino has famously argued that critical discursive interaction provides the very basis for the objectivity of science. While highly influential, CCE has also been criticized for being overly idealized, failing not only as a descriptive but also as a normative account of scientific institutions and practices. In this paper, we examine Longino’s social account of science from the vantage point of a conception of argumentation as epistemic exchange. We show that CCE does not explicitly problematize some important aspects of scientific practices, in particular: the *costs and risks* involved in extensive critical discursive interaction; the imperative of responsible collective *workload management* in a scientific community; and the need for mechanisms of *curation and filtering* in any sufficiently large epistemic community. The argumentation as epistemic exchange model retains the core idea of CCE, namely the centrality of critical discursive interaction in science, but incorporates aspects of scientific practice neglected by CCE (costs and risks, workload management, curation). Our analysis thus adapts CCE to situations where scientists are ‘busy people’ who must contend with limited resources (of time, energy, funding etc.). To illustrate our proposal, we discuss practices of peer review as instances of epistemic exchange. While highlighting the intrinsic vulnerabilities of the peer review system, we also offer some recommendations on how to improve it.

## Introduction

Argumentation, understood as the activity of ‘giving and asking for reasons’ (Brandom 1994) (Bermejo Luque 2011), is a prominent type of human communicative interaction in various domains, notably in law, politics and science. Argumentative processes in these different domains follow general principles, but are also characterized by domain-specific norms. *Science*^1^ in particular is one of our best examples of a disciplined system of collective epistemic activity, and argumentation plays a crucial role in scientific practices.

What is distinctive about scientific discourse, as opposed to discourse in other discursive spheres, is the fact that scientific claims publicly made must be supported by *evidence and arguments*, following communally accepted scientific methods. The bar for persuasion and plausibility is typically set higher in science than in other contexts, and a scientist is required to offer further justification for her claims when challenged by peers (Zamora Bonilla 2006). Indeed, the influential sociologist of science Robert Merton (Merton 1942) identifies what he calls *organized skepticism* as one of the institutional imperatives of modern science: scientific claims should be exposed to extensive critical scrutiny by peers before being accepted.

In this vein, Helen Longino has famously argued that what she calls ‘critical discursive interaction’ provides the very basis for the objectivity of science (Longino 1990) (Longino 2002). Longino contends that the objectivity of scientific knowledge is constituted through norms of inquiry that facilitate transformative criticism among diverse and dissenting peers. The social character of science provides the basis for transformative criticism, which requires adherence to specific socio-epistemic norms. According to her, when scientific research is conducted according to these norms, it becomes objective to the degree that it fulfills these norms. Her account is known as ‘Critical Contextual Empiricism’ (CCE).

Longino’s CCE remains very influential, but it has also been the target of criticism (Solomon 2007) (Biddle 2009) (Steel et al. 2021) (Ajdari 2023). Critics argue in particular that CCE is overly idealized, failing not only as a descriptive but also as a *normative* account of scientific institutions and practices. It has also been argued that the CCE norms can be amended and defended in view of these criticisms (Borgerson 2011). Following Longino’s own recommendation to engage in critical discursive interaction, in this paper we engage with her social account of scientific practice from the perspective of argumentation as *epistemic exchange*, a framework that has been recently developed and applied by one of us and colleagues (Dutilh Novaes 2020b) (Ivani and Dutilh Novaes 2022) (Rittberg 2023). This approach takes inspiration from a sociological framework known as *social exchange theory* (Cook et al. 2013) to develop a model of argumentative interaction as epistemic exchange: exchanges where epistemic resources such as evidence, justification, objections, defeaters etc. are involved. While this approach applies broadly to different argumentative situations, it applies *especially* to science, as a collection of predominantly (though not exclusively) *epistemic* practices.

From this perspective, and drawing on previous research on the costs and risks of critical interaction in science, we argue that the *costs and risks* involved in engaging in extensive critical discursive interaction, both for a scientific community as a whole and for its individual members, must be explicitly and systematically addressed in analyses of scientific practices.^2^ These costs and risks in turn entail that individuals and scientific communities must engage in responsible collective *workload management*, as we’re all *busy people*.^3^ Moreover, we argue that any sufficiently large epistemic community will require *filtering and curating mechanisms* in view of information overload, which in turn create barriers to participation in scientific discourse. These aspects are not explicitly problematized in CCE, and yet the applicability of CCE to the concrete practices of scientists and scientific communities calls for a systematic treatment of them. This is what our epistemic exchange perspective can offer: an account of science that, as CCE, centers critical discursive interaction, but is more realistic insofar as explicitly considers the costs and risks of critical discursive interaction in science, and limitations of time and cognitive resources—in short, *CCE for busy people*. To illustrate our main claims, we discuss practices of *peer review*.

We proceed as follows. In Sect. 2, we present Longino’s social account of scientific practice and objectivity as centered on critical discursive interaction. In Sect. 3, we discuss some of the critiques of CCE in the literature. In Sect. 4, we offer a concise presentation of the conception of argumentation as epistemic exchange, and indicate how it can complement Longino’s account. In Sect. 5, we discuss the peer review system, highlighting some of its intrinsic vulnerabilities but also offering some recommendations on how to improve it.

## Longino’s Millian Account of Critical Discursive Interactions in Science

Longino’s social account of science starts from the familiar idea of underdetermination of theory choice by evidence (Turnbull 2018). She notes that, since empirical evidence can never fully determine whether we should accept or reject a particular scientific theory—there is always a ‘gap’ between data and hypothesis—this means that background assumptions will play a decisive role in theory choice. But background assumptions reflect social, moral, political, and cultural contextual values; thus, scientific theories are not objective in the traditional sense that requires (value) neutrality. According to her, since there is no non-arbitrary way to determine which set of background assumptions is superior, processes of hypothesis evaluation encode implicit (methodological and substantial) value judgments which are not justified, leading to bias in scientific justification.

Longino then goes on to develop an alternative account of scientific objectivity. According to her, scientific knowledge is necessarily a *social* enterprise: it is only when research is produced within a community that the subjective preferences and values of individuals can be transformed into objective beliefs, frameworks, methods, or criteria. This transformation requires that scientists engage extensively in critical discursive interaction, as the ‘clash’ of different perspectives and values allows for a critical evaluation of assumptions.

Longino’s account of scientific practice and scientific objectivity is substantially inspired by John Stuart Mill’s conception of ‘free exchange of ideas’ (Biddle 2009).^4^ In *On Liberty* (1859) (Mill 2012), Mill notes that, when our ideas are challenged by engagement with those who disagree with us, we are forced to critically evaluate our own beliefs.

Man is capable of rectifying his mistakes, by discussion and experience. Not by experience alone. There must be discussion, to show how experience is to be interpreted. Wrong opinions and practices gradually yield to fact and argument; but facts and arguments, to produce any effect on the mind, must be brought before it. (Mill 2012) (p. 38).

According to Mill, this process is beneficial *even* when we are right and our interlocutors are wrong. The expectation is that the remaining beliefs, those that have survived critical challenge, will be better justified than those held before such encounters. Dissenters thus force us to stay epistemically alert instead of becoming too comfortable with entrenched beliefs. Indeed, beliefs become justified (‘deserving of confidence’) through critical engagement:

In the case of any person whose judgment is really deserving of confidence, how has it become so? Because he has kept his mind open to criticism of his opinions and conduct. Because it has been his practice to listen to *all* that could be said against him… (Mill 2012) (p. 39, emphasis added).

Mill seems to be suggesting here that *every single* objection or argument against one’s position must be attended to, even if outlandish or far-fetched, for the belief to become properly justified. (We will return to this point shortly.)

In a similar vein, Longino argues that the exchange of ideas and (genuine) critical discursive interaction is constitutive of scientific knowledge production, understood as a complex web of (mostly cooperative, but occasionally also adversarial) communicative interactions.^5^ As a response to the underdetermination problem, she argues that the solution is to lean on the social character of scientific inquiry, as the different stages of justification are subjected to the critical scrutiny of diverse epistemic groups holding different background assumptions. Critical discursive interactions should allow a community of knowers to uncover their hidden assumptions and biases, re-evaluate them, and transform them if necessary. Longino maintains that, when an epistemic community fosters and cultivates effective transformative criticism among diverse peers, the knowledge produced by the community can be said to be objective, as their assumptions and hypotheses have been critically evaluated. In other words, discursive interactions should counter the undue influence of idiosyncratic biases of an individual or subgroup on which hypotheses are accepted by an epistemic community.

Longino formulates four main community-level norms governing critical discursive interactions, which are supposed to ensure the effectiveness of these interactions:

1. *There must be recognized avenues for the criticism of the evidence, methods and assumptions, and reasoning*

Longino notes that criticism of original research must take place in standard (public) venues such as journals, conferences, and forums where original research is presented. Moreover, critical contributions should be recognized as just as valuable as the original research. She supports this claim with a Millian point: “criticism not only spurs evaluation and reevaluation of hypotheses but also leads to a better appreciation of their grounds and of their consequences” (Longino 2002) (p.129).

Longino acknowledges that many aspects of contemporary science do not suitably support the value of criticism: “The limitations of space, the relation of scientific research to production and commerce whose consequence is the privatization of information and ideas, and an understanding of research as the generation of positive results, all contribute to the marginalization of critical discourse” (Longino 2002) (p. 129). Moreover, while the existence of the Internet has greatly contributed to scientific collaboration across geographical distance (see for example the emergence of ‘crowdsourced mathematics’ (Pease et al. 2020)), these new technologies for communication have also made the *scale* of these scientific conversations much larger, and thus more difficult to manage (a point we will return to later).

(2) *The community as a whole must be responsive to such criticism*

This norm posits that a scientific community should attend to criticism and seriously take it into account. Sound criticism should lead to changes in beliefs and theories held by the community; it should be *transformative*. “The change may comprise the acceptance of different beliefs, the modification of beliefs, the development of new data, reasons, and arguments.” (Longino 2002) (p.130) The same norm should also guide dissenters; they must be responsive to possible rebuttals and must update their criticism in the face of compelling responses.

(3) *There must be shared standards that critiques can invoke*

Not every critical argument should have a free pass; some criticism will simply be irrelevant, misguided, or unsubstantiated (Intemann and De Melo-Martín 2014). There must be ways to distinguish between appropriate and inappropriate dissent, and only appropriate dissent merits substantive engagement—a point which is arguably insufficiently appreciated by Mill, as noted above, and may well only be amenable to contextual solutions. According to Longino, shared standards are relevant to distinguish appropriate from inappropriate criticism.

However, the cogency of a community’s established standards can also be challenged (Lari 2024). These standards are essential to determine in a non-arbitrary way whether a piece of criticism should be taken seriously (or not). Longino suggests that the standards should also be critically scrutinized, thus being subject to change overtime in response to alternative goals or values of inquiry. Just as pieces of knowledge that are justified through critical discursive interactions, these shared norms too are considered by Longino to be the results of critical scrutiny.

(4) *Intellectual authority must be equally shared among qualified practitioners*

A related issue is how to determine *whose* criticisms should be considered authoritative: *who* should be included in or excluded from critical discursive interactions in a scientific community? How is the range of relevant experts and peers who should be part of the ‘conversation’ to be determined?

Longino argues that diversity of perspectives is necessary for effective critical discursive interactions, appealing to the ideal of intellectual democracy. This means that everyone’s contribution should be respected equally. ‘Everyone’ may in principle also include non-scientists, in particular stakeholders (Rolin 2009). However, there are also some constraints to the democratization of intellectual authority. In reality, intellectual authority is distributed unequally: there is a division of epistemic labor, and some of us are endowed with more knowledge on specific topics than others; some count as relevant contributors and some do not. (Institutional factors pertaining to education, credentials, past performance etc. will also be relevant.) For this reason, according to Longino, equality of intellectual authority must be *tempered*, but what exactly this ‘tempering’ amounts to remains underspecified, and may also only be amenable to contextual solutions.

Critical discourse is assumed to be most effective when hypotheses are exposed to the broadest range of criticisms. Longino posits that only the *content* of discourse should matter, not the (social, economic, or otherwise) identity of the dissenters. Every member of a community should be regarded as capable of participating in critical dialogue, irrespective of their social or economic position. For instance, Longino argues that the exclusion from scientific discourse of women, people of color, and scholars working in the Global South is not only socially unjust but it also constitutes a cognitive/epistemic failure. When the critical reflections produced by these groups are systematically excluded from discursive interactions, the racist and/or gendered assumptions of a scientific community are not sufficiently scrutinized and uncovered. This phenomenon is illustrated by various relevant episodes in the history of science (e.g., the gendered studies of human fertility). Longino thus suggests that minority dissent should be actively cultivated by a community not (only) as an expression of justice, but for the sake of epistemic improvement and scientific objectivity. This criterion imposes some “duties of attention and inclusion” (Longino 2002) (p. 132).

These four norms are not meant to be fully descriptive of actual scientific practices. Longino presents them as “features of an idealized epistemic community” (Longino 2002) (p. 134), which can be more or less satisfied by different communities. In practice, their fulfilment is a matter of degrees, but the norms are ideals that epistemic communities should aspire to implement.

## Critiques of Longino’s Account

Thus viewed, CCE seems to presuppose that critical interaction and dissent is *always* beneficial (Intemann and De Melo-Martín 2014)—‘the more, the merrier’ (basically a Millian point). But extensive critical interaction also seems to have downsides, as suggested by the well-known mechanisms of groupthink and polarization (Sunstein 2002) (Broncano-Berrocal and Carter 2021). Moreover, research with computer simulations has shown that scientific communities displaying extensive communication among their members may (surprisingly) be less truth-conducive than less communicative communities (Zollman 2007).

In this vein, Miriam Solomon (Solomon 2007) discusses some representative episodes of belief change in the history of science which, she claims, clash with the CCE norms. She observes that “criticism frequently results in the entrenchment of prior positions (due to pride, confirmation bias, etc.)” (p. 144), as critical deliberative interaction in science is vulnerable to peer pressure, the influence of those in positions of authority, and the silencing of insights and critical evidence. Crucially, Solomon objects that Longino’s proposals are not sufficiently supported by empirical/historical case studies:

[Longino’s] requirements of “equality of intellectual authority” and “responsiveness to criticism,” while sounding reasonable, are not shown to be feasible or effective by application to particular cases. […] In fairness, Longino explicitly regards her account of scientific objectivity as an ideal, which actual cases approach to a greater or lesser degree, resulting in degrees of objectivity. But even this more modest claim is not supported by cases. (Solomon 2007) (p. 45).

Solomon does view dissent in science as epistemically fruitful, but she hypothesizes that dissent *without deliberation* is preferable. Her account is supported by ‘wisdom of the crowd’ considerations—which suggest that a community makes better decisions without deliberation (Surowiecki 2005)—as well as considerations related to Condorcet’s Jury theorem (Solomon 2006). If Solomon is right, then critical discursive interaction actually represents an *impediment* to reaping the benefits of dissent and diversity in epistemic communities.

Another critical reflection on Longino’s norms was recently offered by Steel and colleagues (Steel et al. 2021), based on empirical studies on ‘information elaboration’ in knowledge-producing organizations. Information elaboration (IE) is the process whereby dispersed cognitive resources (such as beliefs, techniques, abilities, heuristics, methods, and insights) are communicated, integrated, and iterated (Steel et al. 2021) (p.1293). Steel and colleagues critically review the empirical findings on how diversity impacts information elaboration, identifying two relevant kinds of diversity for an epistemic community. One is *task-relevant diversity*: diversity in cognitive characteristics that are relevant to the task in question, such as experience, education, expertise, approach, and mastery of methods and techniques. The other is *demographic diversity*: diversity across demographic or social identity-related categories such as gender, ethnicity, religion, geography, ability, race, etc.

Several accounts of the impact of diversity on the epistemic performance of groups emphasize the so-called ‘double-edged sword’ impact of demographic diversity (e.g., the highly cited (Hong and Page 2004)). According to this hypothesis, demographic diversity negatively impacts a group’s epistemic performance because it negatively impacts group cohesion (due to factors such as more conflict, less common ground and less mutual trust between the members of the group). But it also has a positive impact because demographic diversity is often associated with task-relevant diversity, which is widely recognized as favorable for the epistemic performance of groups.

Steel et al. cite empirical evidence that challenges the effective functioning of Longino’s norm of tempered equality, showing that demographic characteristics of critical exchange partners do matter in practice, especially in assuring effective critical interactions. For instance, the hypothesis of ‘cognitive diversity expectation’ suggests that perceived demographic diversity in a group leads group members to expect more cognitive diversity, which in turn leads them to attend to new information more carefully, and assess it more thoroughly to uncover novel insights (Steel et al. 2021) (p. 1294). In demographically homogenous groups, the members expect others to hold similar information and are thus less driven to engage with it critically, potentially failing to benefit from useful dissent. Based on these observations, Steel et al. conclude that “Longino’s model may be of limited use for providing practical guidance or theoretical understanding with regard to actual situations in which mechanisms identified by DBIE [diversity-benefits-information-elaboration] explanations play an important role” (Steel et al. 2021) (p. 1302).

Finally, while Solomon and Steel et al. focus primarily on the descriptive inadequacy of Longino’s account, Justin Biddle argues that Longino’s account is not even *normatively* plausible—at least for epistemic agents who do not have infinite cognitive resources and infinite time and are ‘busy people’. Longino requires that individual agents should in principle be open to *everything*: “A practice of genuinely open criticism and discussion requires an openness to all perspectives: no claim or belief can be held immune to criticism.” (Longino 2002) (p. 159) But agents being open to absolutely every possibility is at odds with the idea of “individuals as intrinsically socially located and thus committed to certain aims, evaluative criteria, and experimental techniques as a result of the fact that they are socialized within certain contexts” (Biddle 2009) (p. 622).

For individuals with such commitments, it may make more sense to pursue their research agendas rather than having to constantly fend off criticism. “The scientists who receive these criticisms are simply uninterested in following them up; they see that as a waste of time and resources and a diversion from the more important task of working out their program.” (Biddle 2009) (p. 621). What Biddle emphasizes (a point which will be crucial for our own analysis) is that critical discursive interaction is very *resource-intensive*, thus diverting valuable cognitive resources and precious time from other, potentially more fruitful endeavors. “Progress in science is best ensured not by demanding of individuals that they be open to everything but, rather, by distributing the resources of a community into various lines of research and letting each of these programs doggedly pursue its own course.” (Biddle 2009) (p. 622).

In the next section, we present an account of argumentative processes understood as epistemic exchange (Dutilh Novaes 2020b). This account allows for a systematic investigation of some aspects of critical discursive interaction in scientific practice that are not explicitly problematized by Longino (but which have been discussed elsewhere in the literature). These are: the *costs and risks* of extensive critical interaction (Lari and Mäki 2024); the imperative of responsible collective *workload management* (Rittberg 2023); and the need *for filtering and curating mechanisms* (also known as ‘gatekeeping’) (Dormandy and Grimley 2024) in any sufficiently large, complex epistemic community where agents must navigate situations of *information overload* (Desmond 2024). Our proposal has the advantage of bringing together these different aspects of scientific practice under one unifying framework. As noted above, an account of the concrete practices of scientists and scientific communities requires a systematic treatment of these aspects. This is what the ‘argumentation as epistemic exchange’ perspective can offer: an account of scientific practices and institutions that centers critical discursive interaction, as in CCE, but which takes into consideration the fact that scientists are ‘busy people’.

## Argumentation as Epistemic Exchange

A comprehensive analysis of critical discursive interactions in scientific practice must take into account not only the cognitive limitations of individual knowers, which require careful individual workload management (Begby 2024), but also the social complexities of these processes. In particular, such an account should specify the conditions under which the positive epistemic effects of critical discursive interaction theorized by Longino are more likely to come about, while also accommodating some of the criticism discussed in Sect. 3. The model of discursive interaction and argumentation that we adopt here recognizes the value of dissent, but goes further in terms of explaining the costs, obstacles, and risks of engaging in critical discursive interaction and identifying favorable conditions for its deployment.

The conceptual backbone of this model is a conceptualization of argumentation as a form of *epistemic exchange*. Importantly, the general idea of viewing social interactions, in particular *discursive* interactions, as instances of exchange is one that Longino herself has articulated:

*Inter*action is a stronger notion than that of *joint* action. We may both attend a concert together. This would be a case of joint action. When we discuss the concert with each other afterwards, however, we are interacting. In our *exchange of ideas*, we are each changed, at a minimum by learning what the other thought of the concert, but perhaps less minimally by modifying our opinion as a consequence of learning of the other’s assessment. […] Whereas joint action involves doing things together and sharing involves holding things together, *interaction involves exchange of some kind* (Longino 2022) (p. 3, emphasis added).

The conceptualization of argumentation as epistemic exchange is inspired by a framework known as *Social Exchange Theory* (SET) (Dutilh Novaes 2020b), developed primarily by sociologists to explain human social behavior as processes of exchange of resources involving costs and rewards, and against a background of social networks and power structures (Cook et al. 2013). SET was originally developed in the late 1950s and early 1960s under the influence of research in economics (rational choice theory), psychology (behaviorism), and anthropology. It combines a first-person perspective, which explains and predicts choices that individuals make between different potential exchange partners, with a third-person perspective, which focuses on structural features of these exchange networks.

From a SET point of view, successful continued exchanges are the very glue of sustainable social relationships between individuals in societies. The key principle is the principle of *reciprocity*, understood in a diachronic sense (I do something for you today, you may do something for me next week), echoing the influential analysis of gift exchange by anthropologist Marcel Mauss (Mauss and Guyer 2016). Moreover, different from economic transactions where resources are primarily goods and services, the resources exchanged in social interactions as conceived by SET can be of different kinds, ranging from symbolic (e.g., recognition, information) to tangible ones (e.g., service, money) (Foa & Foa, 1980).

**Fig. 1:**
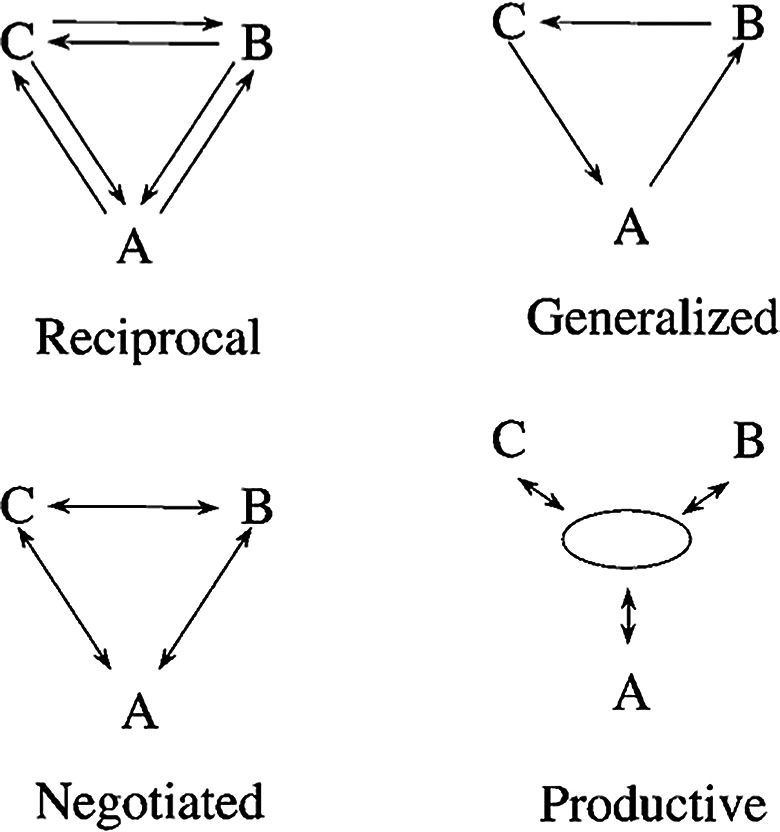
Four forms of social exchange, from (Lawler et al. 2008). The arrows indicate the flow of benefits from one agent to another

One of the SET pioneers, sociologist Richard Emerson (Emerson 1976), introduced an influential distinction between four main types of social exchange (Molm et al. 2007) (see Fig. 1):

- ***Negotiated exchange***: people jointly negotiate the terms of an agreement that benefits both parties, either equally or unequally. Both sides of the exchange are agreed upon at the same time. Commercial transactions are quintessential examples of negotiated exchanges; when the transaction is done, the parties can walk away (as the balance is equal) and may never exchange again. However, if the transaction is successful, the buyer may return to the same supplier in the future.
- ***Reciprocal exchange***: people perform individual acts that benefit others, such as giving assistance or advice, without negotiation and without knowing whether, when or to what extent the other will reciprocate. Reciprocal exchange is rather like a process of gift exchange (Mauss and Guyer 2016), where, overtime, what one gives and what one receives from an exchange partner tends towards equivalence.
- ***Generalized exchange***: the recipient of benefit does not return a benefit directly to the giver, but to another person in the social network. The original giver eventually receives some benefit in return, but from a different person. On the whole, the different people who are part of the system give and receive about the same amount of resources, but in a diffuse way.
- ***Productive exchange***: these are interactions aimed at a jointly produced collective good, where people unilaterally provide benefits to the group and receive benefits from it. Examples of productive exchanges include various kinds of team work and activities, where different people work together to jointly produce something or some outcome, which will benefit all of them.

The insights and results from SET can be applied to epistemic processes, which can be conceptualized in terms of the notion of *epistemic exchanges*: exchanges where epistemic resources such as knowledge, evidence, information etc. are involved. The model allows for a detailed analysis of the conditions under which successful epistemic exchange may occur or fail to occur, in particular in argumentative processes and critical interaction (see (Dutilh Novaes 2020b) and (Dutilh Novaes Forthcoming) for details). Here we focus primarily on two preliminary stages that have significant impact on whether a given agent will be in a position to engage in fruitful epistemic exchange (or not): the *networks* that determine which sources and which epistemic resources an agent is exposed to; and the *contrastive assessments* that agents make regarding the different potential exchange partners.

Thus seen, three distinctive stages for epistemic exchange are worth highlighting:

1. **Attention/exposure**. Social networks determine who is a potential epistemic partner to whom, given the relevant opportunity structures for epistemic engagement. Who is in a position to attract the attention of others? Typically, many signals are being broadcast and various different agents are competing for the receiver’s attention, in a so-called ‘attention economy’ (Wu 2017).
2. **Assessing exchange partners**. Next, individuals assess the potential exchange partners in terms of their suitability. Among those who have caught my initial attention, whom do I deem worthy of consideration for further engagement? At this point, factors pertaining to *trustworthiness* and *prestige* come into play (among others).
3. **Engagement with content**. It is only at a third stage that engagement with *content* properly speaking should occur; this is when the actual epistemic exchange in fact takes place. At this point, the agent will reflectively (and possibly critically) engage with the argument, evidence or content being offered, seeking to understand its substance and evaluate its cogency. The process may lead to a mutually beneficial exchange where all parties involved improve their respective epistemic stances, and in some cases even go on to produce knowledge together (Lakatos 1976).

**Fig. 2:**
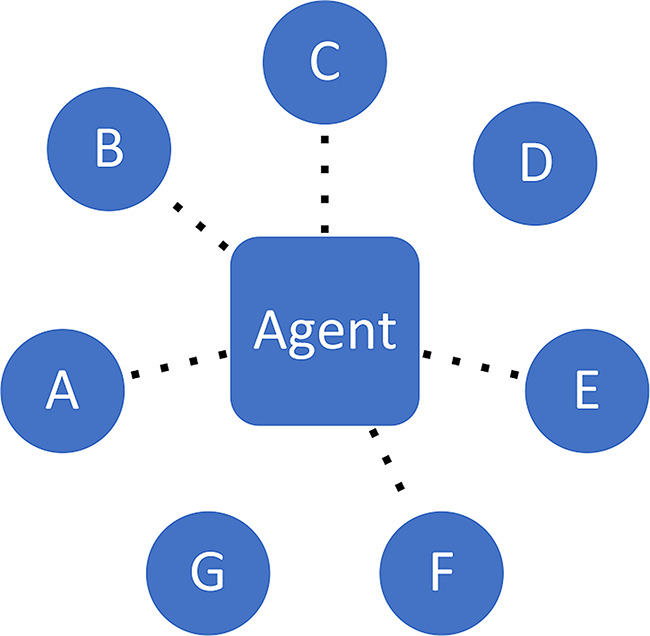
Attention. Agent does not ‘see’ sources D and G, while other sources catch her attention (dotted lines)

**Fig. 3:**
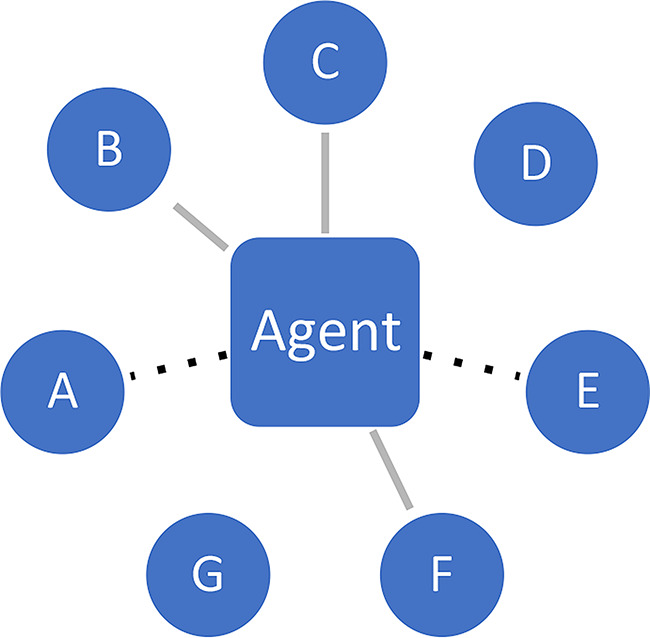
Assessment. Agent deems B, C and F as worthy exchange partners (grey lines), but not A and E

**Fig. 4:**
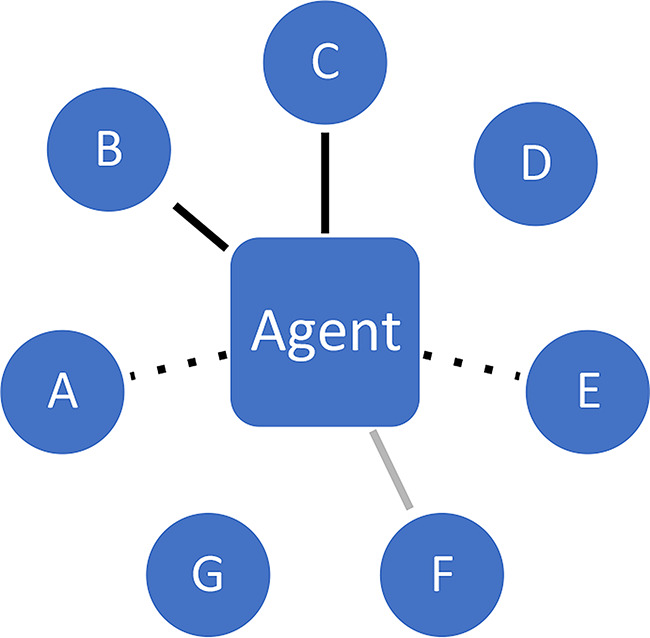
Engagement. Agent eventually engages substantively with B and C (black lines), but not with F (due to, for example, cognitive and time limitations)

Stages 1 and 2 crucially determine if and when someone will seriously engage with the epistemic resources being offered by someone else, following patterns of (unequal) distribution of attention and perceptions of trustworthiness. Figures 2, 3 and 4 represent the three stages.

Furthermore, the epistemic exchange approach considers not only the *benefits* of argumentative interactions but also their *costs and risks* (Paglieri and Castelfranchi 2010). In epistemic exchanges, there are also costs involved for the participants, such as costs of time and cognitive resources. In particular, mounting a thoughtful critique of a scientific position is a demanding task, but the fact that critical engagement is not valued to the same extent in scientific communities as the formulation of novel theories and hypotheses disincentivizes significant expenditure of time and cognitive resources on this activity (a point recognized by Longino and also noted in (Fehr and Jones 2022)). Moreover, there are *opportunity costs* involved in critical discursive interaction: by engaging in such interactions, I will miss out on the opportunity of doing something *else* with my time and resources, for example advancing my own research agenda (Biddle 2009).^6^

There are also significant *risks* involved in critical argumentative exchanges, in science and elsewhere. There are *reputational risks*, for example when a junior scholar dares to criticize a famous researcher. There is the risk of *conflict escalation*, when what starts as a professional interaction between peers turns into a bitter battle (as there is also considerable *power* involved in representing the dominant paradigm in a scientific community). An example of conflict escalation are the so-called ‘linguistic wars’ in the 1960s and 1970s involving Noam Chomsky and his critics (Harris 2021). There is also the risk of *legitimizing problematic positions*: should one really engage critically with positions that are ‘beyond the pale’, for example creationism or racist eugenics? The mere fact of criticizing such views may lend them a dangerous veneer of scientific legitimacy (Martini and Andreoletti 2021). Finally, participants in critical interactions run the risk of becoming perpetrators or victims of *epistemic injustice* (Fricker 2007) (Kidd 2017), when the views and points of criticism proposed by people with social identities that tend to be marginalized in scientific practice (women, people of color, researchers from the Global South etc.) are discounted due to prejudice.

In the next section, we turn to a central component of contemporary scientific practices, namely *peer review*, from the perspective of argumentation as epistemic exchange, so as to illustrate our proposal more concretely.

## Peer Review

Peer review takes place at multiple levels of scientific practice (Lee et al. 2013), including evaluation of research output such as conference submissions, papers, and books; decisions pertaining to research funding, hiring and promotions; and also informally, as critical interaction at conferences, repositories, debates in online communities etc. For tractability, we here focus specifically on pre-publication peer review as solicited by the editors of scientific journals, in its ‘traditional’ form (thus excluding relatively new practices such as pre-registration); but our main points also apply to peer review more generally.

The peer review system is one of the cornerstones of contemporary science, and a key example of a practice through which critical exchanges among peers should improve epistemic outcomes (both for individuals and for communities) (Waltman et al. 2023). It is the embodiment of Mertonian organized skepticism (Huutoniemi 2015), and one of the most important mechanisms for quality control in scientific research (Arvan et al. 2022). And yet, peer review entails extremely *high costs* for a scientific community.^7^ For instance, the total time that reviewers spent on writing peer reviews was estimated in over 100 million hours in 2020 alone (Aczel et al. 2021). The labor that goes into peer reviewing is a precious but limited resource of a scientific community, which must be carefully managed and balanced against the other core tasks of scientists. In view of the need for workload management, filtering and curation mechanisms must be in place, in particular under the responsibility of journal editors.

Peer review is one of the loci of scientific practices where the CCE norms should have normative grip. Academic journals are one of the main *recognized avenues for criticism* in scientific communities (norm 1), showcasing not only original work but also substantive criticism of competing theories. Insofar as the reviews by peers offer constructive criticism and authors improve their papers prior to publication thanks to this criticism, peer review practices (should) embody *responsiveness to criticism* (norm 2). Furthermore, the critiques offered through peer review are expected to reflect the *shared standards* of a scientific community (norm 3) rather than the whimsical opinions of a reviewer. Finally, the idea of *equally shared intellectual authority* (norm 4) is the very foundation for the principle that *peers* are ideally placed to evaluate the quality of the research produced. Additionally, anonymous peer review (which is practiced in many but not all disciplines) is supposed to mitigate the effects of reputation, prestige, geographical origin etc. when the reviewer is evaluating a piece of research, again in the spirit of shared intellectual authority.

However, further reflection shows that peer review, while crucial to scientific research, is a very unstable and vulnerable system. We here discuss two features of the peer review system that make it (intrinsically) vulnerable. Firstly, reviewers are expected to provide vital epistemic resources at a high cost to themselves (cognitive load, time expenditure, opportunity costs), while not getting much in return (Sect. 5.1). Secondly, because journal editors must *defer* to the expertise of peers to make editorial decisions, they will consult peers that they take to be competent and reliable. This means that the pool of reviewers consulted does not (and perhaps *cannot*) reflect the diversity present in the relevant community, thus challenging the norm of shared epistemic authority (Sect. 5.2). Our analysis of the costs, risks and limitations of the peer review system thus illustrates some of the obstacles for the practical applicability of the CCE norms; the ideals posited by CCE stumble upon the reality of scientific practice as performed by busy individuals and strained communities. However, proposed alternatives to the peer review system also present their own issues (discussed in Sect. 5.3), which leads us to conclude that it is still worthwhile to try to improve the peer review system. In closing, we offer some concrete suggestions to this effect.

### Peer Review as a Costly Practice

When solicited by the editor of a scientific journal, a reviewer’s report should contain epistemic resources in the form of constructive criticism and suggestions for the author, which should lead to improvement of their work. But equally (if not more) important, the report should contain an assessment of the merits of the paper for the *editor*, who will then decide whether to consider the paper for publication or not (possibly after rounds of revision). Moreover, indirectly, the whole scientific community benefits from the epistemic resources provided by reviewers, insofar as their critical engagement should help improve research outputs (Andersen 2020) and ensure that only high quality research is published in scientific journals.

However, the system has an intrinsic vulnerability, namely the fact that reviewers are requested to provide vital epistemic resources at a high cost to themselves (cognitive load, time expenditure, opportunity costs) while not getting much ‘in return’. Crucially, peer review for journals is typically conducted *anonymously*, with no financial or immediate reputational gains. As anyone with editorial experience can confirm, securing competent reviews in a timely manner has become increasingly difficult; potential reviewers are themselves overwhelmed by their own immediate obligations, having little time left for such an underappreciated task. The significant costs of producing high-quality reviews coupled with a ‘publish or perish’ culture (which leads to increased numbers of submissions) severely strain the system as a whole, leading to what is now perceived in several disciplines as a ‘peer review crisis’ (Horta and Jung 2024) (Petrescu and Krishen 2022) (Tropini et al. 2023). The ‘replication crisis’ (Romero 2019), in particular in the social sciences, has also been interpreted as a sign that the peer review system does not seem to be doing its job optimally.

Understood as a type of epistemic exchange, peer review instantiates the different types of exchange discussed in the previous section in different ways/to different degrees. Journal peer review is typically not a *negotiated* exchange: reviewers are usually not paid or otherwise tangibly compensated for their work. (The idea of paying for peer review is sometimes floated around (García et al. 2022), but it is not common practice.) As for *reciprocal* exchange, the relation between reviewer and author is not reciprocal, or at least not symmetric: it is primarily the reviewer who provides valuable epistemic resources which are passed on to the author. (In ideal cases, the reviewer can also *learn* something valuable from engaging with the paper, which may be viewed as a form of reciprocation.)^8^ However, the exchange between reviewer and *editor* can take the form of a reciprocal exchange (in a diachronic sense), as the reviewer is doing the editor a favor by reviewing a submission, which may create an informal indebtedness from the editor towards the referee (who may then reciprocate the favor in a different way at a later stage). This may constitute a (mild) incentive for the reviewer to perform the task, or at least a barrier to saying ‘no’ (i.e., to remain in good standing with the editor).

Peer review as a system is perhaps best seen as a form of *generalized* exchange: by design, those involved are *peers*, and thus also *authors* of papers. The thought is that the reviewer’s expenditure of cognitive and epistemic resources will be compensated for when she herself submits a paper to a journal (which may be the same journal or a different one), and receives reviews from peers. This is why it is sometimes recommended that, as a rule of thumb, one should do as many reviews as one submits papers, mutiplied by the number of reports that a paper typically receives (Erlich 2016), which varies per discipline. One should thus receive and contribute roughly the same amount of resources to the peer review system as a whole (‘what comes around, goes around’). Thus seen, one motivation for a researcher to act as a reviewer is being a ‘upstanding citizen’ of a scientific community, on the understanding that this is simply a vital service that every member must provide. But as it stands, the system is not good at catching *free riders*, understood as people who strain the system by submitting many papers while not performing the corresponding duties as reviewers. This is so especially in view of the anonymity of peer review, and the absence of transparent systems to track who does what.

Finally, and more optimistically, peer review can be seen as a form of *productive* exchange. Scientific research is team work, where different people jointly produce scientific findings both by explicitly working together (in scientific teams) and by engaging in (constructive) critical discursive interaction by means of (among others) peer review. An example of such a productive interaction is the peer review process for Andrew Wiles’ famous proof of Fermat’s Last Theorem (FLT), in the early 1990s. The first complete version of the proof contained a serious mistake, which was spotted by dilligent reviewers (Brown 2015). It then took Wiles a full year to fix the proof, resulting in the only proof of FLT to date that is accepted as valid by the mathematical community (Taylor and Wiles 1995).^9^

The idea of peer review as productive exchange seems to come closest to Longino’s account of science as inherently social. However, productive exchange requires a largely *cooperative* background, where the interests of the individuals involved are essentially aligned: they expect that they will all benefit from the outcome of their joint work. At a higher level, it is certainly true that we all benefit from a well-regulated system of knowledge production. But in practice, science also functions in terms of *rivalries and competing interests*; hierarchy and competition are at the heart of academic and scientific practices (Levy 2010) (Dutilh Novaes 2021). Thus, the ideal of peer review as productive exchange is at odds with the competitive incentive structure that is pervasive in scientific research (Hill and Stein 2025).

Fundamentally, the main issue are the meager incentives for busy reviewers to invest their precious time and cognitive resouces on performing reviewing tasks. No immediate benefits accrue, so reviewers have to be motivated by a generalized sense of professional duty, the expectation that they may learn something interesting from the paper, or to ingratiate themselves with the editor. Systems of social exchange function primarily through diachronic relations of reciprocity and accountability, but such relations do not scale up well, for example to large scientific communities. Moreover, anonymity, an important feature of peer review in many (though not all) disciplines intended to compensate for author-based biases, does not mix well with reciprocity and accountability principles, which require more personalized, transparent relations.

In sum, peer review is very costly in terms of the time and cognitive and epistemic resources required from reviewers, both individually and collectively. If members of scientific communities are constantly reviewing the work of their peers, there is the risk of too little time being left for their own research. Scientific communities must engage in responsible collective *workload management*, with a reasonable allocation of resources to critical interaction as well as to the many other facets of scientific research. In particular, some of those who want to be included in scientific discourse and to have their contributions critically engaged with will simply not possess the required credentials, for example so-called ‘cranks’ in mathematics. The community as a whole cannot afford to expend considerable resources on low-quality research. 10

### Biases in Editorial Processes

It is widely acknowledged that peer review is vulnerable to various problematic biases, pertaining both to author and reviewer characteristics (prestige bias, nationality bias, language bias), as well as to content (confirmation bias, conservatism, bias favoring positive results) (Lee et al. 2013). Biases in peer review can appear at multiple levels, but here we address specifically editorial practices in scientific journals.^11^

The three tiers of our model of epistemic exchange shed light on the phenomenon of bias in peer review, especially as pertaining to editorial decisions. Typically, when an editor receives a submission, she will first decide whether the paper deserves to be sent out to reviewers or should instead be desk-rejected. In journals that work with a triple-anonymous system, editors are not informed of the identity of the author, and thus must make this first decision based solely on the merits of the paper. But most journals, at least in philosophy, still operate with a double-anonymous system, and knowing the identity of the author (their prestige, nationality etc.) will typically influence the editor’s estimation of the paper’s potential. (In mathematics and related fields, the reviewers are typically also aware of the identity of the author(s).)

If the editor does decide to send the paper to reviewers, the next step is *reviewer choice*. Naturally, the editor will only consider those who are in her network of potential exchange partners (tier 1); these are the scholars whom she is aware of as working on the relevant topics. But of course, visibility in these networks will be unevenly distributed: prominent people in the field, or people whom the editor happens to know (for institutional or other reasons), will more easily come to mind (an instance of the *availability heuristic* (Tversky and Kahneman 1973)).

Next, the editor will consider the various possibilities and make choices that reflect her assessments of *the competence and trustworthiness* of potential reviewers (tier 2). Who is sufficiently competent on the relevant topics to evaluate the arguments presented in the paper? Who can be trusted to ascertain whether the paper constitutes a novel and interesting contribution to the literature? Who can be trusted to produce an impartial report, not overly colored by their own preferences and views? Will they deliver the report in a timely manner? Here, the biases of the editor will fundamentally affect her choice of reviewers, and it is not evident whether these biases can or even *should* be fully mitigated. After all, the editor will be *deferring* (Levy 2022) to the reviewer’s expertise and judgement, so she needs to have sufficient *trust* in the reviewer. And of course, reviewer choice will crucially determine the paper’s chances of eventually being accepted for publication.

Moreover, the burden of performing reviewing tasks will not, and cannot, be evenly distributed within the community. There are individual discrepancies between the ‘upstanding citizens’ who go above and beyond their duties to the community and the ‘free-riders’ who systematically decline reviewer requests with no negative repercussions to themselves (while also having their own papers peer-reviewed). But there are also variations pertaining to *levels of seniority*. A (reasonable) rule of thumb that most journal editors seem to follow is that only those with PhD degrees qualify as journal reviewers; exceptions can be made for advanced PhD candidates, but it is not common.

Thus, specific members of a scientific community, in particular those who have already obtained their PhD degrees, are saddled with a more substantial reviewing burden. In the current ‘publish or perish’ climate, PhD candidates are encouraged to submit papers to scientific journals to boost their job market prospects; they add to the *demand* side of the peer review system while contributing only limitedly to the *supply* side. Moreover, fairness considerations also dictate that academics in precarious situations (temporarily employed or unemployed) should be burdened to a lesser degree by the reviewing needs of the community, so that they can spend more time and energy in career-furthering activities (something that peer review certainly is not). As a result, the reviewer pool, and in particular the pool of reviewers who are *de facto* frequently consulted, will most likely *not* reflect the real diversity of views, skills and values in the relevant scientific community. This pool will be skewed towards more prominent members of the community, and will privilege the orientations and research programs endorsed by the journal editors (Katzav and Vaesen 2017).

There is also a well-known geographical imbalance: researchers from wealthier countries have significantly wider representation in the reviewer pool (Zumel Dumlao and Teplitskiy 2023). Since reviewers (and editors) will tend to favor work that is similar to theirs (following the principle of homophily (McPherson et al. 2001)), geographical imbalance in reviewer pool will tend to increase acceptance for publication of research produced in wealthier countries, especially in North America and Western Europe.^12^ In other words, the so-called ‘Matthew effect’ (Merton 1968), whereby ‘the rich get richer and the poor get poorer’, seems to be one of the consequences of peer reviewer selection.

These observations suggest that Longino’s ideal of scientific objectivity as emerging from wide, inclusive, critical discursive interaction is not optimally supported by the current journal peer review system. And while at least some of these issues may be counterbalanced by suitable design interventions, the effects of attentional networks and of perceptions of trustworthiness can simply not be fully mitigated; they are arguably *constitutive* of the structure of epistemic exchanges, especially in large-scale communities. Indeed, the issues pertaining to peer review and editorial choices just discussed are not merely practical issues related to implementation: they are intrinsic to any sufficiently large epistemic community that must contend with different kinds of gatekeeping, filtering and curating practices (Dormandy and Grimley 2024). As noted by Solomon (Solomon 2007) (p. 45), “no one has a realistic model for a scientific community in which inequalities in intellectual authority do not play a large role”, but this feature of scientific communities is in tension with Longino’s norm of shared intellectual authority.

In large-scale groups such as contemporary scientific communities, it is simply not feasible for all members to engage with the work of all others (as is the case perhaps in smaller scientific communities, for example learned societies in the 17^th^ and 18^th^ centuries). In communities where sizable amounts of research are produced, specific individuals and institutions must be in charge of curating the output, thus being in a position to influence what is perceived as certified research and what gets paid attention to (or not). Indeed, this is essentially a *scale problem*: when a scientific community grows beyond what individual members can keep track of themselves, filtering and curation mechanisms become indispensable. Gatekeepers such as journal editors must rely on heuristics to manage the allocation of epistemic resources within a scientific community, especially attention and certification, which will inevitably leave room for biases.

### Alternatives?

Given these issues with the peer review system, the question arises: are there alternative models for scientific publication? Some fields (such as mathematics, physics and computer science) rely heavily on pre-print repositories like ArXiv. While it may seem that such repositories will avoid the exclusionary effects of (pre-publication) peer review, in practice they too must rely on filtering mechanisms to establish what may be placed in the repository or not, and crucially, in which category within the repository the paper will be placed. “ArXiv thus functions as a certification site, by dividing its population into insiders and outsiders in the eyes of the wider physics community.” (Reyes-Galindo 2016) (p. 599–600) Enthusiasts of the repository model (Arvan et al. 2022) (Heesen and Bright 2021) tend not to sufficiently appreciate their limitations, for example the uneven distribution of attention and the barriers to innovation in these platforms. Indeed, in the pre-publication peer review system, a paper that passes a first quality control and is sent to peer review will be reviewed by at least one (and typically more) peers, even if the author is very junior or from a non-prestigious institution. By contrast, in repositories, expenditure of epistemic resources in the form of (informal) peer review (i.e., critical engagement) will tend to concentrate on those who already have a high standing in the relevant community (another instance of the Matthew effect).

Another alternative might be to limit or even eliminate gatekeeping in editorial processes (perhaps in a Feyerabendian spirit of ‘let a thousand flowers bloom’). But in recent years, a ‘natural experiment’ of what happens when journal editors do not perform curating duties rigorously has been taking place in the form of *predatory journals*, following the ‘pay-to-publish’ model (Richtig et al. 2018). These journals tend to have a very low bar for acceptance (going as far as accepting *all* submissions that they receive), since they make a profit from each publication; the peer review process, if it happens at all, does not properly filter out papers of poor quality.^13^ As a result, the problem of information overload (Desmond 2024) is exacerbated: scientific publishers are now producing more papers than ever before, severely straining the scientific publishing system (Hanson et al. 2024). Low-quality work published in predatory journals then acquires a veneer of respectability, which in turn causes epistemic confusion, especially among laypeople but also among scientists themselves (Richtig et al. 2018). Predatory journals thus show that curation and gatekeeping remain essential for scientific communities.

Of course, the dysfunctionalities of academic publishing do not occur in a vacuum: they occur in the context of a ‘publish or perish’ climate and severe competition for scarce opportunities such as jobs and funding, and against the background of greedy practices by commercial academic publishers. It is impossible to truly reform the scientific publication system while these adjacent factors remain in place, which in turn reflect broader economic, cultural and political circumstances. However, pre-publication peer review, while being an intrinsically unstable and bias-prone system, may well be the ‘least bad’ of all alternatives (much like democracy, as famously claimed by Winston Churchill).

This leads us to consider possible interventions that may make the peer review system less vulnerable.^14^ Our epistemic exchange perspective suggests some concrete recommendations on how to improve the system, thus addressing the question of the conditions under which the kind of critical discursive interaction recommended by CCE is more likely to be fruitful.

- The incentive structures for peer review should be modified, so as to make it a fairer ‘exchange’ for the reviewers. Platforms for peer review recognition such as Publons already exist, but for now still have limited impact. A wider, unified system for tracking the peer review contributions of individual academics may help alleviate the free-rider problem and make accountability more transparent.
- Junior scholars (PhD candidates) should receive formal training on how to perform peer review. Such training should reflect the shared standards of the relevant scientific community (in the spirit of Longino’s norm of shared standards), and should facilitate and homogenize the process of peer review.
- Journals should make conscious efforts to hire editors and editorial associates from underrepresented groups in academia, in particular scholars from the Global South, who will be in a better position to ensure a more diverse pool of reviewers.
- Journals should invest more in *managing* peer review, by hiring editorial assistants and paying editors. (We do not think that paying *reviewers* for their work is likely to make a significant difference.) Especially commercial academic publishers most certainly have the resources to offer fair compensation for these services.

## Conclusions

In this paper, we engaged critically with Longino’s CCE account of critical discursive interaction in science. We showed that CCE does not explicitly problematize some important components of scientific practices, in particular: the *costs and risks* involved in extensive critical discursive interaction; the imperative of responsible collective *workload management* in a scientific community; and the need for mechanisms of *curation and filtering* in any sufficiently large epistemic community. Ultimately, a scientific community needs mechanisms to ascertain which arguments and evidence are worth engaging with and which ones are best ignored; it is neither feasible nor desirable to engage with them all. CCE rests on a highly idealized vision of agents who do not have to contend with limited resources. As such, its practical applicability to the finite, busy agents that we are is limited.

To address these limitations of CCE, we presented a model of argumentation that, as CCE, emphasizes the value of dissent and critical interaction for science, but goes further in terms of explaining the costs, obstacles, and risks of engaging in critical discursive interaction within scientific practice. (In fairness, Longino tacitly or explicitly recognizes many of the issues that we identified here pertaining to critical discursive interaction in practice.) Drawing on social exchange theory, the model centers the concept of *epistemic exchange* to explain epistemic processes, and allows for a more realistic account of them as processes where agents and groups are constantly engaging in workload management. To illustrate our analysis, we investigated the peer review system of academic publishing. We argued that this system is vulnerable and unstable, but that alternative models for academic publishing seem even more problematic. We closed with some (modest) recommendations on how to improve the peer review system, in the spirit of CCE for busy people.

## Footnotes

1 For the purposes of this paper, we understand ‘science’ in a broad sense, encompassing not only the natural and life sciences, but also the social sciences and the humanities—essentially, the whole range of epistemic activities covered by the German term ‘*Wissenschaft*’.

2 In other words, we propose an account of scientific practices that takes onboard the general idea that agents must make choices within constraints, in particular against the background of limited supply of resources, which agents and communities must carefully manage. In this respect, our investigation falls broadly within the framework of *bounded rationality* (Grüne‐Yanoff, 2007).

3 We borrow the term ‘busy people’ from the book *Democracy for Busy People*, by political scientist Kevin J. Elliott (Elliott 2023). Elliott develops an analysis of what democratic institutions should be like so as to allow for participation of people who simply don’t have the time or energy to engage in demanding democratic processes, in view of caring obligations and the need to provide for their families.

4 Biddle uses the phrase ‘marketplace of ideas’ to refer to Mill’s position. We prefer ‘free exchange of ideas’, as the logic of the market is different from the logic of exchange (Graeber 2014). Moreover, the notion of a marketplace of ideas is arguably not a good description of Mill’s actual position (Gordon 1997).

5 Mercier and Sperber (Mercier and Sperber 2017) (Chap. 18) offer a similar argumentative, interactionist account of scientific practice.

6 Note that, while Biddle focuses on the costs involved in the *uptake* of criticism, we here focus primarily on the costs involved in *formulating* criticism.

7 There are many other issues with peer review and the academic publishing system which we do not discuss here, such as the absurd profit margin of commercial academic publishers and paywalls for research produced with public money, among others.

8 More generally (and perhaps cynically), while laborious, the work of peer review also confers a certain amount of *power* to the reviewer, for example in terms of supporting or else undermining specific investigations or research programs by other (competing) researchers.

9 See (Dutilh Novaes 2020a) (Chap. 11) for a detailed analysis of such productive exchanges in mathematics.

10 As argued in (Rittberg 2023), while seemingly unjust, the fact that professional mathematicians largely ignore (presumed) cranks can be justified by workload management considerations. See (Hodges 1998) for some examples of ‘cranky papers’ submitted to mathematical journals.

11 Some fields rely heavily on pre-print repositories like ArXiv (primarily for mathematics, physics and computer science) instead of pre-publication peer review. We comment on this model below.

12 The stark geographical inequalities in research have many causes, including variability in availability of research funding and other institutional factors. But the fact that most reviewers are based in wealthy countries in North America and Western Europe at least partially explains the abysmal rates of articles published in scientific journals involving authors based in the Global South (e.g., just 2,7% in a selection of natural science journals between 2015 and 2022 (Baker 2023)).

13 A related, very worrisome recent phenomenon is the existence of *paper mills*: organizations that produce low-quality, plagiarized or fake research papers and sell authorship (Christopher 2021). These papers then often appear in predatory journals.

14 See (Copeland and Marin 2024) for a complementary approach on how to reform the peer review system, specifically in philosophy.
